# ‘HighChest’: An Augmented Freezer Designed for Smart Food Management and Promotion of Eco-Efficient Behaviour

**DOI:** 10.3390/s17061357

**Published:** 2017-06-11

**Authors:** Manuele Bonaccorsi, Stefano Betti, Giovanni Rateni, Dario Esposito, Alessia Brischetto, Marco Marseglia, Paolo Dario, Filippo Cavallo

**Affiliations:** 1 The BioRobotics Institute, Scuola Superiore Sant’Anna, Viale Rinaldo Piaggio 34, Pontedera 56025, Italy; manuele.bonaccorsi@santannapisa.it (M.B.); stefano.betti@santannapisa.it (S.B.); giovanni.rateni@santannapisa.it (G.R.); dario.esposito@santannapisa.it (D.E.); paolo.dario@santannapisa.it (P.D.); 2 Laboratory of Ergonomy & Design, Department of Architecture, University of Florence, Via Sandro Pertini 93, Calenzano 50041, Italy; alessia.brischetto@unifi.it; 3 Laboratory of Design for Sustainability, Department of Architecture, University of Florence, Via Sandro Pertini 93, Calenzano 50041, Italy; marco.marseglia@unifi.it

**Keywords:** smart freezer, food management, eco-efficient behavior, energy saving, waste reduction

## Abstract

This paper introduces HighChest, an innovative smart freezer designed to promote energy efficient behavior and the responsible use of food. Introducing a novel human–machine interface (HMI) design developed through assessment phases and a user involvement stage, HighChest is state of the art, featuring smart services that exploit embedded sensors and Internet of things functionalities, which enhance the local capabilities of the appliance. The industrial design thinking approach followed for the advanced HMI is intended to maximize the social impact of the food management service, enhancing both the user experience of the product and the user’s willingness to adopt eco- and energy-friendly behaviors. The sensor equipment realizes automatic recognition of food by learning from the users, as well as automatic localization inside the deposit space. Moreover, it provides monitoring of the appliance’s usage, avoiding temperature and humidity issues related to improper use. Experimental tests were conducted to evaluate the localization system, and the results showed 100% accuracy for weights greater or equal to 0.5 kg. Drifts due to the lid opening and prolonged usage time were also measured, to implement automatic reset corrections.

## 1. Introduction

Roughly one-third of the edible food produced for human consumption is lost or wasted globally, which is approximately 1.3 billion tons per year [1]. The impact of food waste is not only financial, but also environmental and social. Indeed, it involves gas emission, oil degradation and energy consumption, in addition to the paradox related to food access difficulties in some less developed areas. Food losses in industrialized countries are as high as those in developing countries; however, in the latter more than 40% of the food losses occur at post-harvest and processing levels, whereas in industrialized countries, more than 40% of the food losses occur at retail and consumer levels. The amount of food waste at the consumer level is 222 million tons, nearly as high as the total net food production in sub-Saharan Africa (230 million tons) [2]. For this reason, the promotion of consumer eco-efficient behavior and an effective, easy-to-use professional and household food management system are very important for reducing the extent of food loss.

A recent survey concerning food waste in Italy [3] revealed that 48.2% of the wasted food had passed the expiration date, whereas 36.7% and 11.5% had been left respectively in the fridge or pantry for too long. In addition, 11.5% of waste was due to errors in meal planning and purchasing. From the same survey, a possible solution for the reduction of food waste emerged, since 46.5% of respondents said that intelligent refrigerators or cupboards would help them in planning an optimized shopping list, and determining what is in the house and what is about to expire. Suggestions to improve the use of domestic food resources were also identified: some respondents considered it helpful to receive recipes for reusing leftovers, many would have liked to get advice on how to best preserve their own food, and several said they would like to receive information on the freshness and life of the product. Most users also stated they would prefer to use modern technology to receive such information, such as e-mail and dedicated apps on mobile devices.

Motivated by the socio-economic problems discussed above and taking advantage of the survey recommendation described above, the current work depicts in detail a set of smart services deployed to enhance the functionality of a domestic chest freezer, in terms of food management, eco-efficiency and energy saving. The features implemented, which aim to reduce food and energy waste, are designed not only for the domestic environment, but also for the commercial and catering field, as the system is easily scalable. The promotion of eco-efficient behavior and smart food management is fundamentally the inner intelligence of the system.

Chest freezers are usually under a cost-driven manufacturing process, to deliver low-cost products in the mainstream markets. HighChest thus aims to drive the introduction of Internet of things (IoT) and advanced design to turn traditional chest freezers into advanced appliances able to induce eco-friendly and socially sustainable user behaviors.

## 2. Related Works

Chest freezers come in a wide range of sizes, ranging from approximately 1.5 cubic meters up to 8 cubic meters. They take up more space, but remain more energy efficient than upright and refrigerator freezers, thanks to their horizontal configuration. In particular, chest freezers are more energy-efficient because cool air does not rise very quickly and therefore does not escape every time the lid is opened [4].

Smart systems for food management in household appliances are usually available for refrigerators, but few freezers perform food management or food quality assessment. To the best of the authors’ knowledge, research concerning smart freezers has mainly focused on upright freezers for domestic use. The authors found no research concerning advanced food management or promotion of eco- and energy-friendly behaviors in relation to chest freezers, either for private or for professional use. Gu et al. [5] developed a smart fridge system to provide a well-balanced eating habits recommendation service. The system monitors food currently inside a refrigerator via radio-frequency identification (RFID) tags and a reader. The fact that the proposed system requires RFID tags is a downside, however, as such tags are rarely included in local regulations or provided with food packages available on the mass market. Luo et al. [6] also focused on healthy nutritional habits, introducing a system for shopping lists and recipe recommendations, based on food stored in the refrigerator and users’ dietary profiles. The system uses a local database storing information on users’ nutrition habits, weight, height, age, medical record and allergies, among other things. It is equipped with a barcode reader for the tracking of products and a touch screen on the fridge’s door that acts as a user interface. Rouillard [7] proposed a smartphone-based inventory management solution, which allows users to remotely manage food stocks. The software recognizes food through barcode scanning using cameras or by users’ vocal inputs using a speech recognition service. Additional services are provided, including reminders of expiration times of food in both the fridge and in cupboards sent using SMS and instant messaging.

The RFID Smart Fridge proposed by Noutchet [8] is a modified fridge that includes an RFID scanner tracking all food transactions and providing reminders of product expiration, automatic shopping list generation and order replenishment. Unfortunately, these services require RFID tags on each food package. Sandholm et al. [9] realized a system based on a fridge-embedded webcam to leverage Google Search By Image services to allow automatic recognition of foods. The system aims to improve acceptance and reduce the obtrusiveness of the food recognition process, leveraging the use of cameras. Food recognition is based on the identification of logos and text on packages, which suffers from point-of-view constraints. Sandholm et al. also proposed an automatic item position detection using an infrared (IR) proximity sensor embedded in the freezer compartment.

Murata et al. [10] proposed an eco-feedback system to save power by detecting the opening and closing of the fridge door. The refrigerator is instrumented with internal and external IR distance sensors and a magnetic door switch sensor, to recognize opening and closing events. External IR sensors detect human approaches to the fridge, while internal IR detects food presence in specific positions.

Investigating the Espacenet Patent Data base, the US patent No US 2012/0278190 [11] and the application patent No WO 2016/098124 [12] claim the use of display interfaces on the appliance and tablets and smartphones for the direct or remote control of the refrigerator. Commercial appliances nowadays allow accessing the food database, control and managing expiration dates and alarms or performing on-line shopping or access media. Among the commercially-available smart refrigerators, it is worth mentioning the Samsung Family Hub™ [13], which is provided with a wide touch screen and dedicated Apps for on-line food shopping, media streaming, advanced user touch, and vocal interaction. The LG LFX31995ST smart fridge [14] is instrumented with a tablet for food freshness tracking, inventory, family water intake tracker and daily recipe suggestion. In some cases, cameras are used to see the fridge content, while embedded electronic interfaces allow the managing and setting of alarms in case of power supply fault or food expiration. The LG Smart ThinQ™ Refrigerator [15], instead, embeds an 8-inch Wi-Fi LCD screen that serves as a control panel for the refrigerator, as well as an information hub and potential family organization center. The Smart ThinQ™ provides energy efficiency, a family water intake tracker and daily recipe suggestions. Nevertheless, all of the previously mentioned food management services require the manual input of information from the users. For example, describing and labeling all food, and imputing expiration dates are completely manual procedures. A dedicated Android and iOS application makes the inventory and product expiry data remotely accessible, and a ZigBee radio device enables communication with third-party smart plugs for energy monitoring.

These kinds of freezers are connected to Internet using wireless communication like Wi-Fi and GSM, in the IoT framework, to deliver alarms and information on the inner food stored, directly to the users by means of App on smartphone or tablets. Innovation often regards the process for the food recognition, the organization of a database, the technology and processes for the management of expiring foods. Nevertheless, smart systems for food management were found only for refrigerators and there are no smart chest freezers in the IoT framework. Taking in account for the current state of the art, as in Section 2, we propose the HighChest freezer as an IoT device for advanced food management and the promotion of eco-friendly behaviors. We do believe that a smart freezer with advanced user interfaces and services for the automated food and appliance management would effectively promote a responsible use of the food and energy.

According to the current literature, many open issues remain concerning the development of highly acceptable and effective smart freezers able to improve food management, and to promote eco-efficient and socially sustainable user behaviors. A comparison between the main reviewed existing smart fridge systems and the device proposed in this work is presented in Table 1. Comparison is only performed in relation to refrigerators, since, to our best knowledge, smart freezer technologies do not exist in literature.

## 3. Materials and Methods

### 3.1. Requirements and Design Evaluation

Food and space management, and user interface were accurately considered and analyzed to enhance the product’s user experience and the user’s willingness to adopt eco- and energy-friendly behaviors. Anthropometry, biomechanics and cognitive aspects were carefully taken into account in the first stage of appliance design. Indeed, a virtual simulation process was used to collect relevant data concerning user–appliance physical interactions, and to assess ideal product dimensions in order to maximize product accessibility. In the simulations, anthropometric features belonging to the 5th, 50th and 95th percentiles were considered. Makehuman™ and Rhinoceros software was used in this phase. The Task Analysis (TA) method was followed [16] to investigate the activities related to the user experience. TA is a type of expert assessment that is commonly used to evaluate and validate existing or under-development products. Using this method, it is necessary to identify the critical issues and the design requirements. The main objective was to evaluate the whole product design and the control panel, in relation to macro-activity management and maintenance. Task identification performed for the use of the product is fundamental for investigating and addressing potential criticisms, and obtaining new dedicated product design solutions (see expert evaluation, Table 2).

Based on the results obtained from expert assessment, a number of tests with real end-users were performed (see user trials, Table 2). Gender, provenance, habits, capacities, lifestyle and knowledge of the product were also considered. A total of 11 users attended the sessions, including six men and five women aged between 35 and 75 years. The sessions were held within an area where typical domestic freezer use conditions were recreated. The objects of the evaluation were freezers present on the market, particularly two models commercialized by Whirpool, the SPACE MAX 300 L and 214 L. Users were involved in experimentation using a hybrid method of investigation, simultaneously developing Contextual Inquiry [17], Observation and Thinking Aloud [18], following a heuristic approach. Contextual Inquiry refers to a method for collecting information concerning the interaction between the user and the considered product/system, and, subsequently, mapping out a more or less uniform schedule of the significant aspects to be considered in the synthesis phase. The Observation is conducted by monitoring users during the execution of activities, taking notes of what happens. Finally, Thinking Aloud is a method that requires the subjects to speak aloud while they are solving a problem or performing a task. The test session with users was conducted by considering a Life Cycle Design approach [19], with reference to resource minimization. In contrast, the use phase, underlining the most impactful factors in terms of energy consumption and food waste, was based on the basis of the product life cycle. Particularly the observation was conducted monitoring users during the execution of activities, taking notes of what was happening. In particular, the subjects were asked to store and pick-up from the test bed freezers a number of different objects, representing specific food typologies including fish, meat, vegetables, fruit, and bread. The users’ behaviors were observed and registered, to assess the proper placement and the performed actions. The users posture and pose were also observed, to evaluate the most appropriate ergonomic design for the HighChest prototype. Furthermore, the user-friendliness with technologies was assessed by means of dedicated questions on the use of electronic devices, appliances, smartphones, and Apps. The users were asked to describe how do they use or would use a chest freezer, and to evaluate their interfaces, intuitiveness, user-friendliness, and utility, along with their functionality and utility.

The main objectives of the test sessions can be summarized as follows:

- check the accessibility parameters of the HighChest;
- observe when and how users employ the product in a real environment;
- evaluate usability features of the product and interface;
- assess if users adopt eco-efficient behaviors;
- check if users adopt customized solutions for food storage and management over time;
- find possible correlation between users’ eating habits and storage modalities;
- assess users’ needs and expectations;
- check the level of users’ acceptance of future technological implementations of the system.

### 3.2. Functional Performance Evaluation

The functional performance evaluation of the system was tested on the proposed novel food localization system (see details in Section 6). The aim was to investigate the ability of the system to autonomously locate and weight food while stored, without requiring extensive food labeling for example with RFID tags or requiring expensive or intrusive wireless technologies. The testing of the localization system was performed to assess (i) the food positioning accuracy for an easy retrieval and (ii) the weight fluctuations due to air humidity intake. The food localization accuracy directly affects the time spent by the users seeking for the desired food, and thus the temperature and energy loss due to the lid opening. Humid air is responsible for frost stratification and increases the chest weight, generating bias on the food weight measures.

1. Positioning accuracy: each load cell has been calibrated using a certified sample weight of 1 kg. Then six different samples of 0.1 kg, 0.2 kg, 0.5 kg, 1.0 kg, 1.5 kg and 4.0 kg have been used to assess the localization accuracy of the system over four different positions for each region. For each known position, it has been then calculated the localization error, in the form of Euclidean distance between measured and real x and y.
2. Humid air intake: an experimental procedure has been performed, in order to measure the humid air intake, measuring the freezer weight variation keeping the lid open. Humid air enters in the fridge every time the lid is open for food insertion or retrieval, and generates frost on the inner surfaces, improving the chest weight and reducing the energy efficiency of the freezer, due to the low thermic conductivity of ice. The inner temperature was set to −25 °C, while the room temperature and relative humidity were respectively +22 °C and 30%. The worst case of empty freezer was selected, to maximize the internal/external air exchange. A continuous weight measurement was performed starting with the lid close and ending one minute after lid opening. The measure was repeated 10 times to have a significant data set.

## 4. Design Phases Outcome

The outcomes of the assessment phases and user involvement stage provided information concerning user–product interaction and users’ needs. Critical aspects and solutions regarding food and space management, and interface design are listed in Table 3. These outcomes drove the prototype design proposal and the technological developments later described in this paper. The improvements carried out were oriented towards smart food and space management, and the development of a more effective and intuitive interface, in order to allow conscious space management of the stored foodstuffs, promoting eco-efficient use and reducing waste related to improper storage.

Particularly, the HighChest was conceived to provide (i) a service to identify food and suggest the user the proper storage position to maximize the quality of preservation; (ii) an advanced 3D food localization system to minimize food retrieval time and provide a conscious storage; (iii) a reminder service for food expiration and recipe suggestion to prevent food waste; (iv) energy efficiency in the use of the freezer by facilitating the food retrieval thanks to the embedded food localization system and by monitoring the door opening.

## 5. Description of the System

**Figure 1:**
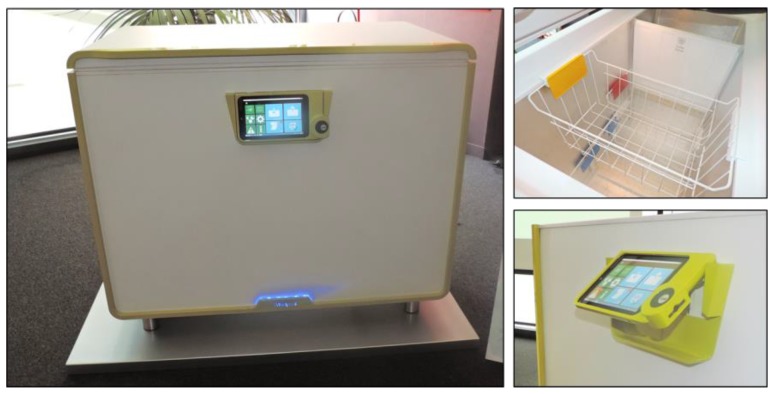
The final HighChest smart freezer prototype, showing details of the inner space and user interface.

We achieved the design and realization of a working prototype of the HighChest freezer (Figure 1). In terms of sustainability, this prototype included recycled materials such as polyethylene terephthalate (PET) in its plastic parts and utilized polyurethane (PUR) from renewable sources for the insulating material.

### 5.1. Description of Hardware

**Figure 2:**
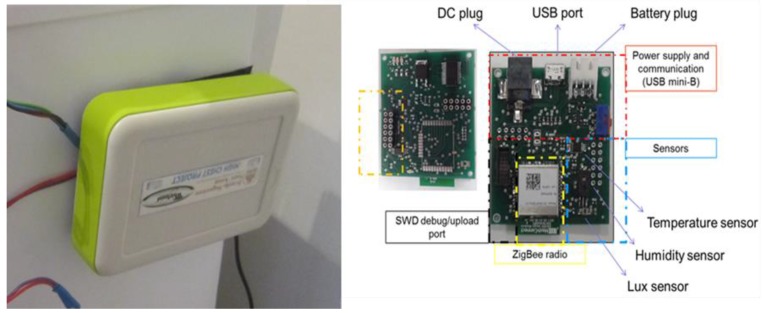
Sensor Board detail from first concept prototype, as well as an architectural description.

HighChest was built using a commercial chest freezer (Whirlpool Space Max CO25OW) instrumented with five electronic modules fixed to the chest’s exterior, requiring few hardware modifications. The system included:

1. Tablet. A commercial 10” tablet to run the graphical user interface for chest management and provide audio-video feedback to users (Figure 1).
2. ZigBee Data-Logger (Data L). The data logger provided ZigBee connectivity to the commercial tablet to gather data concerning freezer status from the connected low-power wireless sensors.
3. ZigBee sensor board (Sensor B). A wireless sensor board was instrumented with two temperature sensors, one for the inner and one for the outer temperature, as well as a humidity sensor and a reed switch to monitor the status of the door (Figure 2);
4. Integrated scale. An outer scale system was integrated into the chest’s adjustable feet. The scale system is composed of four load cells, and a signal amplifier and conditioning system. The scale is USB connected to the tablet.
5. Barcode reader (BCr). A barcode reader was integrated into the chest’s front panel and connected to the tablet using a USB connection.

All the data were collected through the HighChest’s USB connected Data-L. In particular, the ZigBee dongle created a low-power wireless network connecting the remote ZigBee sensor board, allowing the system to gather temperature, humidity and door status data (see Table 4).

**Figure 3:**
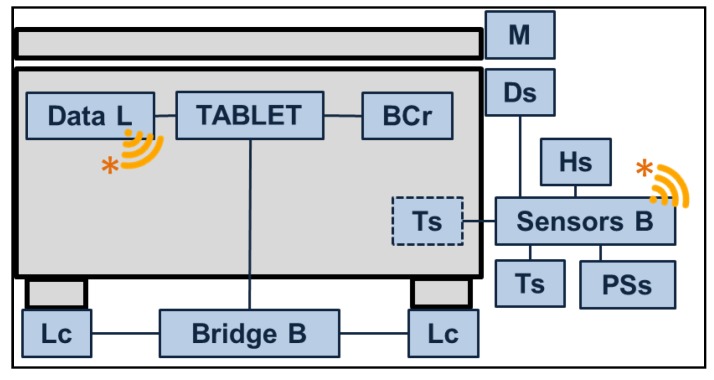
Hardware architecture of the sensor- and HMI-augmented chest freezer. The system included an electronic board (Sensors B), connected to internal and external temperature sensors (Ts), a humidity sensor (Hs), a magnet (M) and sensor (Ds) for door status detection, a circuit for monitoring power supply (PSs), a data logger electronic board (Data L), a tablet acting as HMI for the system (TABLET), a barcode reader (BCr), and four load cells (Lc) and their acquisition board (Bridge B).

Load cell signals were pre-processed and conditioned by a PhidgetBridge from Phidgets (Calgary, AB, Canada). The load cells were installed under the freezer’s feet to obtain an external measure of the chest’s weight. This avoided the need to conduct any installation inside the refrigerated storage, under high-humidity and low-temperature conditions. The MCR12 CCD barcode scanner from ChampTek (Taipei, Taiwan) provided EAN8/EAN13 compliant barcode readings to the tablet. The overall hardware architecture of HighChest is shown in Figure 3.

### 5.2. Graphical User Interface (HMI)

**Figure 4:**
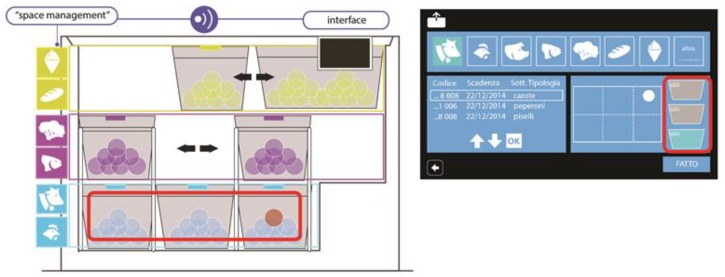
HighChest and graphic interface initial concepts.

The evaluation stage and, most importantly, the test session carried out with users drove the concept design of both HighChest and its graphic interface (Figure 4). The application of the TA method together with concepts from interaction design, visual design and information architecture allowed the identification of interface requirements and usability parameters [20] necessary for user-friendly management of the task flow, which was required for interaction with the system.

Results suggested the use of a touch screen implementing a simple and intuitive user interface for food and space management. The interface would guide the user through the operations of food insertion and withdrawal, improved by a food positioning service described later in this paper. TA outcomes suggested providing certain information to the users, including:

- The appliance status: information concerning inner temperature and alarms would be provided to the user through the graphic interface, as well as remotely, using internet services such as e-mail sending.
- The space management status: the system would provide an easy way to identify the most suitable position in which to store each typology of food and the most easiest method of withdrawal. Audio and visual feedback would improve the chest’s usability, and the user would also be able to manually set the food position according to his/her preference.
- Expiration dates of food: food expiration alarms would be provided using the interface and internet services, and an overview of the expiration dates would be provided using text or visual feedback.
- The promotion of eco-efficient behavior: recipes would be addressed to the users using audio/video feedback, to promote and facilitate the use of food close to expiry.

**Figure 5:**
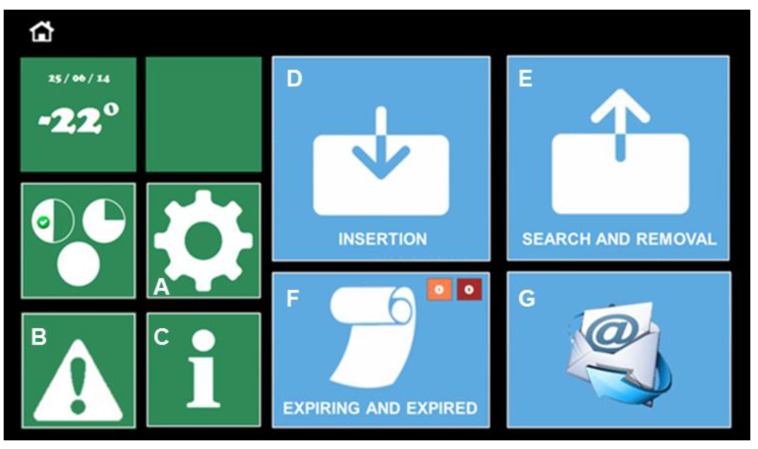
User interface home screen on the HighChest smart freezer. Refer to main text to explanations of each label.

Each of the implemented features can be exploited through the software interface available on an integrated tablet (Figure 5). The main screen is composed of two macro-areas, the chest status management area (Section 5.2.1) in green on the left and the food management area (Section 5.2.2) in blue on the right. Each colored macro-area contains input controls (buttons, text fields, checkboxes, dropdown lists, list boxes, toggles, date field), navigational components (search field, pagination, slider, tags, icons) and informational components (tool tips, icons, notifications, message boxes), which are enhanced by visibility and natural mapping parameters. To respect the principle of functional and perceptive coherence, whenever reminders, tips or advertisements are needed, they are reported to the user with specific pop-ups. Orange indicates products close to expiration, whereas red indicates those that have expired.

#### 5.2.1. HighChest Status

This section consists of a pane that provides date and inner temperature, as well as three buttons that give access to:

- setting date, time, language and email address (Figure 5A);
- status parameters and alarms (Figure 5B);
- information related to technical support, such as phone number and user manual (Figure 5C).

#### 5.2.2. Food Management

This section consists of four buttons that lead to:

- the item insertion process (Figure 5D);
- the item search and/or withdrawal process (Figure 5E);
- the chest inventory, and warnings of expiring and expired products in orange and red, respectively (Figure 5F);
- the option to instantly send of an email containing all information concerning the chest’s status and its contents (Figure 5G).

### 5.3. Inventory Creation Service

**Figure 6:**
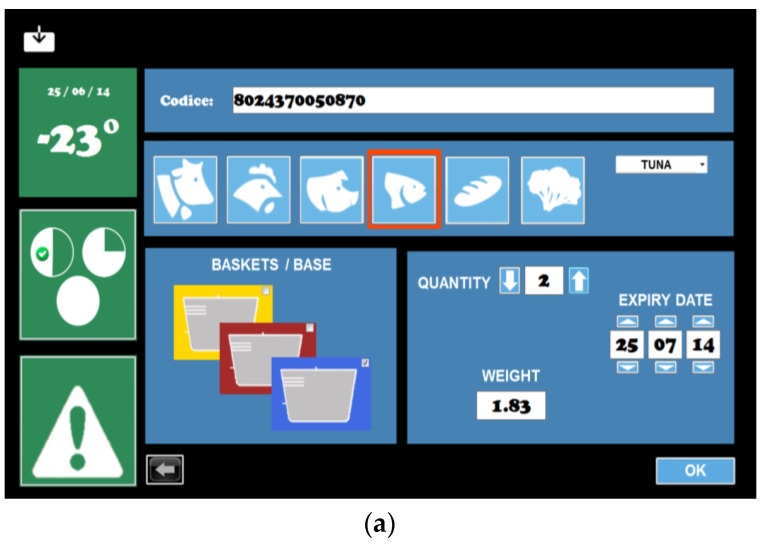

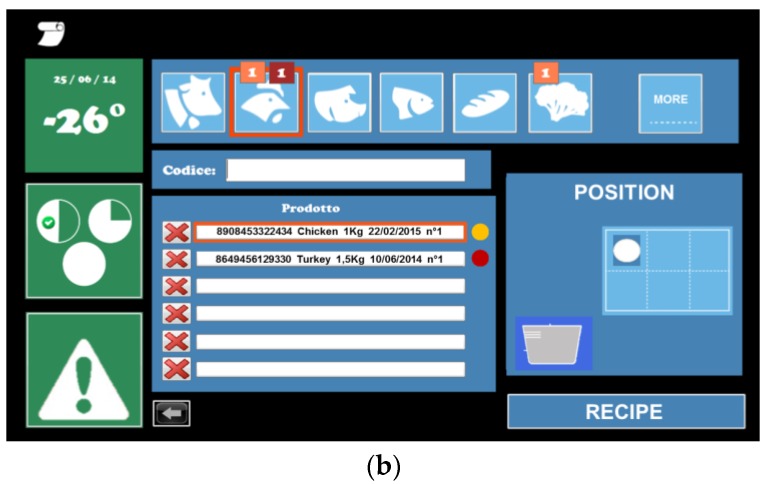
Interface on HighChest freezer prototype, displaying (**a**) product insertion and (**b**) food inventory.

**Figure 7:**
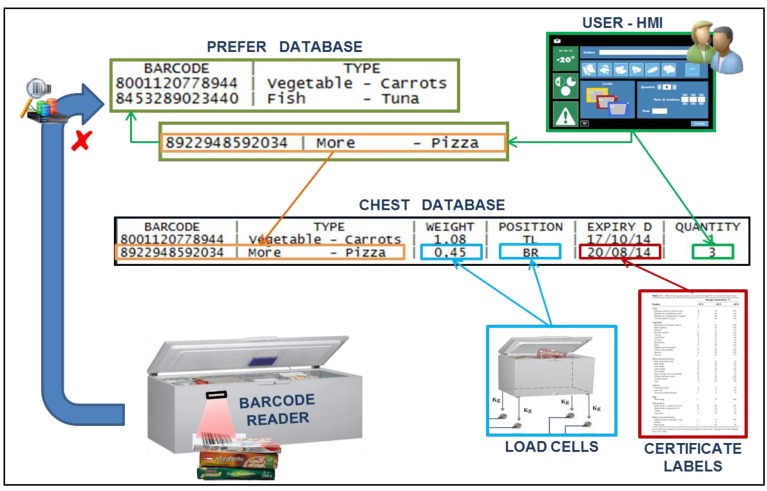
Product registration and the creation process of the two databases.

The inventory was created in a semi-automatic way with user contribution, and contains information concerning barcodes, types of food available, expiration dates, and food weight and position in the chest freezer (Figure 6). Two databases were created to implement the required functionalities: “Prefer” and “Chest”. The first one represented the historical memory of the chest, combining any barcode, known from the chest, with the type of food it represents. The second one was the inventory of stored food in that specific moment. The databases and their implementation are shown in Figure 7.

**Figure 8:**
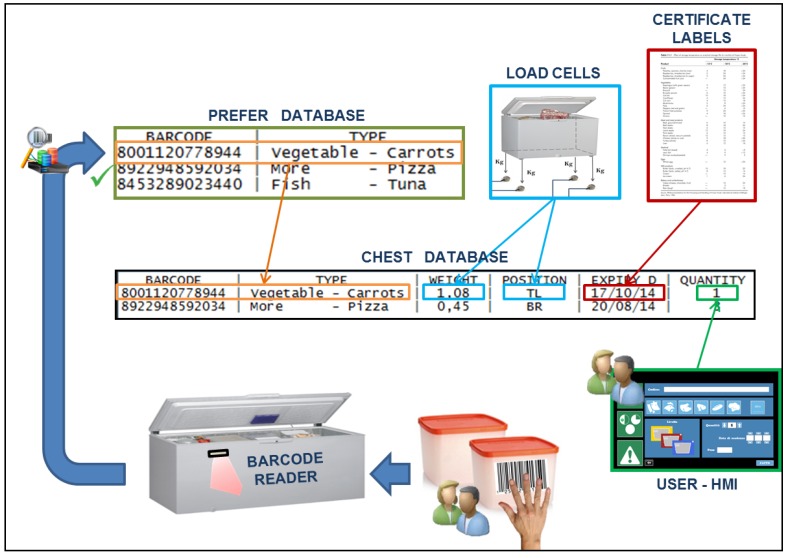
Semi-automated procedure related to the insertion of a product with a barcode already known by the HighChest freezer. The “type” information is taken from the “Prefer” database.

During the registration of a new product (barcode not previously recorded), after reading the barcode, the user must indicate the type of food through the software interface. Indeed, a lack of information regarding the type of food and the expiration date makes the barcode an incomplete method of data retrieval [21]. Only the nation of origin, the manufacturer and the trade name of the product are specified within the code, implying that a single barcode univocally identifies all identical commercial products; therefore, single product features have been combined with the barcode in specific databases via software/user. This information forms the “Prefer” database, also copied in the “Chest” database. The freezer system automatically calculates the date of recommended consumption using certified charts [22]. All this new information is stored in the “Chest” database, combined with food barcode and type. The information remains in the Chest database until the selected product is removed by the user. The “Prefer” database is updated only if insertion is performed and the information is not erased when the product is removed. Thus, the entry process is completely automated if the same product is inserted a second time, because of the same barcode. When the barcode is known to the chest, not being a first registration, the type information will not be derived from the user, but will instead be taken from the “Prefer” database. The procedures set to record a product become completely dependent on the system, and the user must only place the product near the barcode reader at the beginning and confirm data registration at the end (Figure 8). For homemade or unpackaged purchased products, standardized barcodes are provided with the chest, depending on the type of food, to be applied to the preserving containers.

#### 5.3.1. Expiration Reminder Service

The expiry date, combined with the barcode in the “Chest” database, is automatically calculated by the system, which adds the number of days suggested by certified tables to the entry date [22]. If the expiration date is specified on the food packaging, the user can change it manually during the registration process. Once a day, the system checks the database and notifies the user if a product is close to expiration. The user can set a threshold after which the food is defined as “expiring”. A visual alert on the chest interface and an e-mail notification remind the user to consume the expiring products, avoiding the waste of food. If a product exceeds its expiration date without being consumed, it is moved to the expired list. The user is encouraged to not consume the expired food and to remove it.

#### 5.3.2. Store Management

When planning meals or preparing for grocery shopping, the user can check the list of frozen foods stored in the freezer. The navigation within the “Chest” database is mainly based on the type classification. The user can select the requested type of food through the interface and verify the presence of a selected kind of product, its quantity and weight. The user can also have access on the list of stored item from remote, through the periodic sending of mail, to one or more preset mail addresses. The user can go to the food market knowing what he needs or plan his meal without going home and opening the chest freezer. The user can easily delete the product and its features from the “Chest” database using HMI, but not from “Prefer”. This action clearly must follow the actual removal of the product from the chest.

#### 5.3.3. Item Localization

Organization of the inner space is one of the main problems with chest freezers. Searching for a product inside the chest is difficult, especially if it has been frozen for a long time. The user must manually move many products to look for the desired one, without knowing where it is located. We solved this problem by implementing an innovative strategy for space management and food localization. The inner chest volume was divided into three vertical sections, each of which corresponds to a different storage temperature, suitable for preserving different types of food (e.g., fish, vegetables). In the first two levels, the localization of the products is made possible by the presence of baskets that are movable and allow the user to freely shift them. These baskets facilitate food withdrawal, minimizing the time required to find food.

During food insertion, the user is advised by the HMI on the most suitable basket in which to store that specific food, and can then select on the graphical interface the vertical position of the basket where the food will be placed. Moreover, this architecture simplifies access to frequently used items. If the user places a product in the freezer base, in addition to its weight, the system automatically detects and memorizes the horizontal section affected by the load. The location is shown on a two-dimensional map. Specifically, the chest base area is divided into six rectangular subdivisions: Button Left (BL), Top Left (TL), Button Centre (BC), Top Centre (TC), Button Right (BR) and Top Right (TR). Once an inventory is created, the product list, accessible via user interface, is composed of a sequence of buttons, through which the user can browse the stored products’ characteristics and their locations.

#### 5.3.4. Recipe Suggestion Service

When the user selects a stored product via the interface, in addition to the related information, the system presents recipes based on the choice. Thus, the cooking process is assisted by written or video guides. If there are products that have passed the expiring threshold, when the user selects a product, the system provides recipes combining the selected item with the expiring products.

#### 5.3.5. Status Monitoring Service

**Figure 9:**
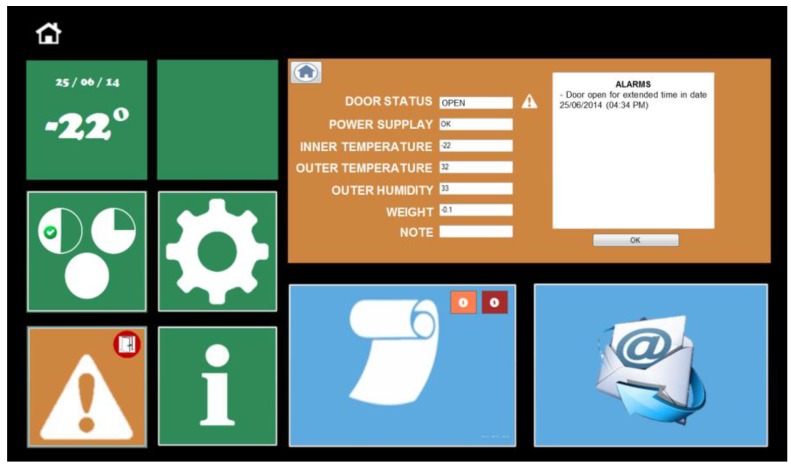
Chest status screen, with system parameters and alarms.

A set of sensors monitors the working condition of the chest, providing warnings if the evaluated parameters reveal the presence of a malfunction. In the case of a malfunction, the system alerts the user through the interface (Figure 9) with colors and warning symbols, and through the LEDs located on the front panel, which have the two-fold role of aesthetics and alarm function (changing color). When the door remains open too long, the chest system points out this not eco-efficient behavior or advises the user if the open door status is not voluntary. Thus, the user can remove the mechanical impediment or remedy their forgetfulness. In addition to avoiding high unwanted energy consumption, this alarm can prevent raising the freezer temperature and causing defrosting. Temperature increases can be generated by many factors, such as an abnormal functioning of the compressor or power failure. This is the reason why an alarm is implemented upon establishing an inner temperature threshold. If the temperature signal overtakes −18 °C (maximum temperature guaranteed by the class of chest freezer) [23], a specific temperature alarm is shown in the status panel of the interface. The temperature alarm triggers a timer that is stopped when the inner temperature returns to the normal range. If the timer reaches the threshold, a second alarm kicks off to signal that foods are thawing. This is an important alarm for the users’ health, because it will notify them if products have been thawed and refrozen, and refrozen products represent a threat for human health [22]. Furthermore, the battery present on the tablet permits it to advise the user when the power source of the chest freezer is temporarily missing. This information, as previously described, can be sent from the chest to the remote device of the user by e-mail and/or SMS. The user can personally intervene or warn someone else in order to avoid the progressive increase of the temperature and potential thawing.

### 5.4. Localisation System Test

The localization system is designed to weigh and locate objects in the entire storage volume. A smart scale system is used to detect position on the horizontal plane, using data from four load cells. Food positioning can be improved to three dimensions using the HighChest space management system. The user places food in the three-level baskets, available at three different heights from the base. Thanks to this information, the localization system is able to retrieve the objects’ positions in the 3-D inner space, improving the ease of food retrieval. According to the space management system design, the chest’s base is divided into six regions of 28 cm × 23 cm. First, 2-D localization is performed using the Weighted Centroid Localisation algorithm [24], which provides X-Y position estimation (*P_x_* and *P_y_*) of a new inserted food item from the weights measured by each load cell (*w_i_*) and the coordinates of each load cell in the freezer spatial reference system (*P_i_*(*x*) and *P_i_*(*y*)). The estimated position is then associated to the relative sub-area identified in the design phase, and the result is displayed to the user, giving an intuitive location assessment.

$$\begin{array}{cc} \begin{array}{c} P_{x}=\frac{\sum_{i=1}^{4}w_{i}\cdot P_{i}\left(x\right)}{\sum_{i=1}^{4}w_{i}}\\ P_{y}=\frac{\sum_{i=1}^{4}w_{i}\cdot P_{i}\left(y\right)}{\sum_{i=1}^{4}w_{i}} \end{array} & \left\{ \begin{array}{c} \mathrm{if}\;P_{x}\leq 28\;\mathrm{and}\;P_{y}\geq 23\to \mathrm{Compartment}\;1,\\ \mathrm{if}\;28<P_{x}\leq 56\;\mathrm{and}\;P_{y}\geq 23\to \mathrm{Compartment}\;2,\\ \mathrm{if}\;P_{x}>56\;\mathrm{and}\;P_{y}\geq 23\to \mathrm{Compartment}\;3,\\ \mathrm{if}\;P_{x}\leq 28\;\mathrm{and}\;P_{y}<23\to \mathrm{Compartment}\;4,\\ \mathrm{if}\;28<P_{x}\leq 56\;\mathrm{and}\;P_{y}<23\to \mathrm{Compartment}\;5,\\ \mathrm{if}\;P_{x}>56\;\mathrm{and}\;P_{y}<23\to \mathrm{Compartment}\;6 \end{array}\right. \end{array}$$

## 6. Results

The localization system was tested to assess (i) food positioning accuracy for easy retrieval and (ii) the weight fluctuations that occur due to air humidity intake. Food localization accuracy directly affects the time spent by users seeking the desired food, thus indirectly affecting the temperature and energy loss due to the lid being open. Humid air is responsible for frost stratification and increases the chest weight, generating bias in relation to food weight measures.

**Figure 10:**
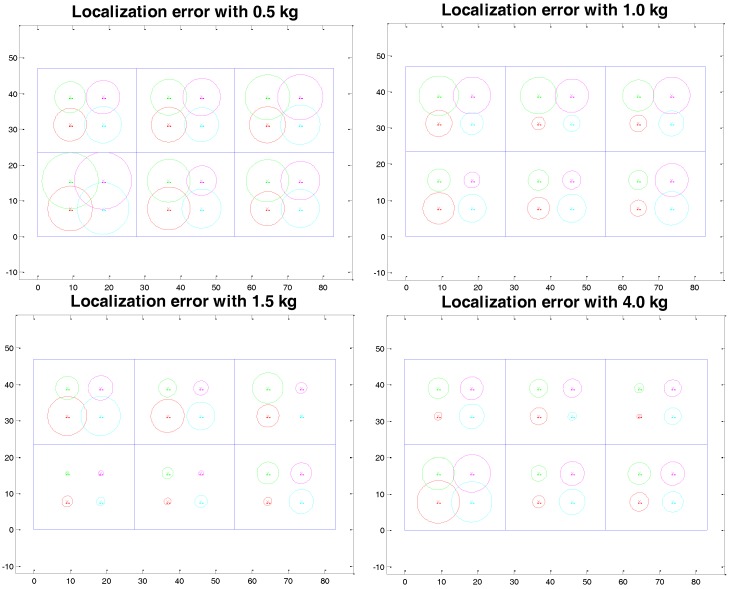
Localization error at four points in each of six different compartments for four different object weights. Each error circle was plotted using the Euclidean distance of x and y position errors $\left(\sqrt{{x_{err}}^{2}+{y_{err}}^{2}}\right)$ as the radius. All errors decreased with increasing weight.

Each load cell was calibrated using a certified sample weight of 1 kg. Six different samples of 0.1 kg, 0.2 kg, 0.5 kg, 1.0 kg, 1.5 kg and 4.0 kg were then used to assess the localization accuracy of the system over four different positions for each compartment (each sample tested 24 times). For each known position, the localization error was calculated, in the form of the Euclidean distance between measured and real *x* and *y* coordinates. This difference was more pronounced for small weights and decreased with increasing weight. The average localization errors among all compartments were 22.94 ± 2.49 cm, 9.42 ± 4.18 cm, 5.60 ± 0.93 cm, 3.60 ± 0.43 cm, 2.43 ± 1.27 cm and 1.87 ± 1.26 cm for weights of 0.1 kg, 0.2 kg, 0.5 kg, 1.0 kg, 1.5 kg and 4.0 kg, respectively. In Figure 10 and Table 5, the error is presented for weights more than 0.5 kg, for which the success rate in identifying the right compartment was 100%. In the case of 0.2 kg and 0.1 kg, the success rate in identifying the right compartment was respectively about 96% and 50%.

**Figure 11:**
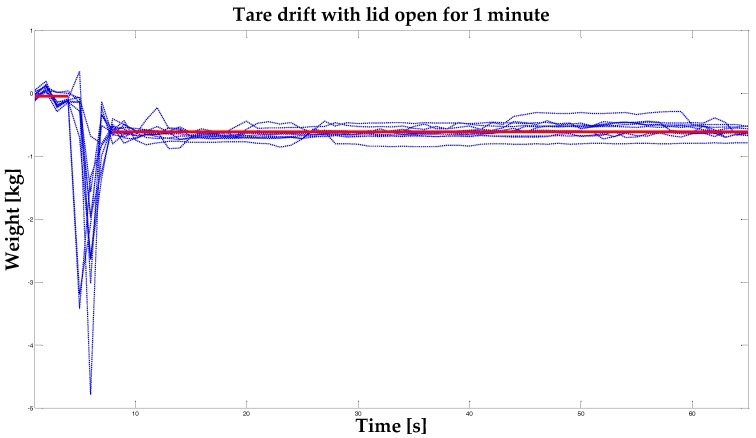
Tare drift continuously measured from 5 seconds before opening of the freezer lid to 1 min after lid opening, for 10 repetitions. The pre-opening weight was −0.0509 ± 0.0023 kg and the post-opening weight was −0.6146 ± 0.0071 kg. Neglecting the transience due to the effect of manual opening, the average drift due to opening was −0.56 kg.

An experimental procedure was performed, in order to measure the humid air intake, in turn measuring the freezer weight variation when keeping the lid open. Humid air enters in the fridge every time the lid is opened for food insertion or retrieval, and generates frost on the inner surfaces, increasing the chest weight and reducing the energy efficiency of the freezer, due to the low thermic conductivity of ice. The inner temperature was set to −25 °C, whereas the room temperature and relative humidity were +22 °C and 30%, respectively. The worst case of empty freezer was selected, to maximize the internal/external air exchange. A continuous weight measurement was performed, starting with the lid closed and ending one minute after the lid was open. The measure was repeated 10 times to achieve a significant data set. Neglecting the transience due to the effect of manual opening, the average bias due to opening was −0.56 kg (Figure 11). This bias was used to filter out the effect of humid air intake on food weight estimation using the integrated scale.

## 7. Discussion

The smart functionalities previously described were implemented in a working prototype. The aim of our work was to embed capabilities for food management and energy saving in a domestic chest freezer, overcoming limitations found in current technology, which is so far available only in the field of augmented refrigerators. Expert evaluation and user trials were conducted by a multidisciplinary team, composed of designers, researchers and expert engineers from Whirlpool, to extrapolate key design points. These outcomes drove the prototype design proposal and the technological developments. The improvements carried out were oriented towards smart food and space management, as well as the development of an effective and intuitive interface, in order to allow conscious space management of the stored foodstuffs, promote eco-efficient use and reduce waste related to improper storage. In terms of sustainability, the developed prototype was also beneficial due to its utilization of recycled materials (such as PET) for its plastic parts and its application of PUR from renewable sources for its insulating material.

Some reviewed works proposed alternative tracking strategies in place of barcodes [3,6,7,8] because of the lack of usability and narrowness of information offered by barcodes. Nonetheless, the use of barcode technology allows the exploitation of the food packaging labels present on the majority of European Point of Sale (POS) commercial products. The employment of a pre-existing code and a low-cost barcode reader makes this codification preferable even to radio-identification (RFID) technology. Indeed, this experimentation demonstrated the opportunity to provide a believable food localization service using a low-cost scale system, instead of RFID and localization system, that requires food tag labeling. The food box tagging with RFID systems is a promising technique, for the simultaneous localization and identification of goods. Nevertheless, local regulations do not impose such technologies in the food/packaging industry and, in the majority of the world countries, only barcodes are used for the identification of food categories. The proposed localization system aims to empower smart appliances with advanced food management capabilities, independently from the presence of smart tags on food, with good positioning accuracy. If, in the near future, RFID tags are commonly used to encode and monitor food, this technology could be integrated into our system to replace or complement the barcode. On the other hand, Sandholm et al. [7] proposed automatic item position detection using compartment-embedded IR proximity sensors. No low-cost solution based on sensors within the room is feasible at freezer operating temperatures. For this reason, we opted for the choice of using load cells under the chest’s feet. With this solution, we can simultaneously achieve the weighing of inserted products and their horizontal localization. The main purpose of our system is to identify the correct insertion quadrant for each product. Our test results, described in the previous section, show that for 0.5 kg, 1 kg, 1.5 kg and 4 kg, 100% accuracy of localization was achieved. Furthermore, Sandholm et al. and Murata et al. [7,8] equipped a refrigerator door with sensors to know when the door closes or opens, which they respectively used to detect wasteful usage of a fridge and to know when video frames should be captured and analyzed. We used a magnetic switch in combination with temperature sensors and a power supply detector to realize a high-level functionality able to warn the user of unintentional prolonged openings, monitor rising temperatures and, in the case of detected thawing, alert the user to not consume refrozen foods. Furthermore, the battery present on the tablet advises the user when the power source of the chest freezer is temporarily missing. Another innovative feature was implemented in our system, leveraging the combined use of door status magnetic switch and load cells to detect lid-opening events, which produce slight variation in the tare. The implemented functionality automatically resets the tare at each lid-opening event. The variation amount calculated after a drift test is used, in order to implement automatic tare reset. In addition, the weight control is also used to produce a warning if the user leans on the chest during an insertion procedure.

Load cells are subject to creep over time, as reported in the producer’s datasheet. This lead to a drift in the weight measure that could be significant for long-time installation and during the appliance life. In our system this problem is overcome, by using the load cells to perform differential measurements. Every time a new food is inserted, the system is recalibrated using weight data registered before and after the lid opening. This method allows to exploit measured food weight as a differential measure on the whole content weight, for the limited time period of the lid opening, filtering out the sensor drift due to the creep.

The proposed system can locate the food placed on the freezer floor, as well as the food stacked on or placed in the baskets. The positioning algorithm assesses the barycenter of the new inserted food, and is unbiased by the presence of other items above or under the new inserted item. Indeed HighChest (as the majority of commercial chest freezer), has the compressor engine mounted on the right side, under the freezer floor. The localization errors accounted for the compartments affected by the presence of the compressor were comparable to the errors accounted for the other compartments (see Table 5), demonstrating that the presence of items or the level/elevation of the food do not affect the localization service accuracy.

Additionally, the size of baskets is appropriate to cover the majority of food products in the market, thus enabling us to speculate that the likelihood to place very large packages, out of the boundaries of the baskets, is very low. It is clear that for large packages, for which it is necessary to remove one or more baskets, the localization system stops to correctly work, or, at least, the system can work only for the remaining baskets. Indeed the freezer design was intended to improve the food management, including the minimization of the reordering actions. The use of inner baskets, dedicated to store specific types of food, was intended to facilitate the inspection, retrieve and placement of food, without the need to remove items from the freezer. It means that the current working conditions, with defined and optimized size of baskets, should drive users to not perform displacements and changes in the position of products.

Eventually, the localization system is continuously sensible to any variation of weight in a differential mode, i.e., it is able to measure the variation of weight in relation to the activities that are performed. These activities include not only the food placement and retrieve, but also possible temporary displacements to facilitate another product placement or when the user moves the baskets along the rails for inserting food into the baskets located at the bottom. Of course, we are aware that possible “unconventional” usage could occur, such as temporary or permanent displacement of the food already stored or large size food placement. In this case, further improvement of the system will be required. In this sense, the freezer reordering procedure could be improved by the introduction of new technologies, like RFID tags. Radio tags would allow the simultaneous identification and localization of the stored food. However the localization and identification performance of this technology is nowadays problematic, as said before, because of the electromagnetic reflections of metals like the inner aluminum coating in the freezers that should be further investigated, and of the lack of standardized use of RFID on food packages.

The connectivity capability of HighChest makes it particularly suitable to be considered as an Internet of Things (IoT) device, able to improve the usability and utility of smart-freezers by sharing knowledge of foods and allowing the remote control of the appliance. The Inventory creation service was designed to be implemented on each single appliance. The chest can generate new entries in the “prefer database” or retrieve information from it either if that database runs locally on the machine, or is provided as a cloud service or hosted on a web server. In the latter case, the HighChest freezer acts as an IoT connected device able to share fundamental information for the improvement of the appliance usability and utility. Indeed, the sharing of information about a barcode reading, the associated food typology and the weight, among all the connected smart freezers, will enable the collective automatic learning of food features. This will produce a significant time saving for the users and a progressive procedure simplification for the recognition of new food typologies. The status monitoring service could be also provided in the IoT framework, connecting each user with the associated freezer. The individual access of the HighChest IoT devices to the “preferred database” and the analysis of the triggered food-expiration, temperature, food-defrost or door-opening alarms would also provide useful information for the assessment of the eco-friendly behaviors of the connected users. This could contribute to the estimation of the annual food waste and the refinement of food-waste prevention or eco-friendly awareness campaigns.

With an IoT oriented deployment, data security becomes a crucial topic. Indeed, ensuring privacy and personal data protection in the IoT framework is fundamental [25], since smart appliances multiply the points of entry and processing for personal/sensitive data and their automated decisions, based on personal data, could directly impact on the users. Privacy risks include the opportunity that a smart freezer could provide private information about the users eating habits, revealing in some cases food allergies or intolerances. To prevent this happening, the web server or cloud platform providing the proposed HighChest IoT service manager, would have a public-key certificate. The connection with each freezer would also take place in a secure channel, implementing for example a Transport Layer Security (TLS) or a Secure Sockets Layer (SSL) protocol. Another crucial issues is the protection of the sensitive personal information (SPI), that can be used by third-part software or unauthorized entities, to identify, contact, or locate the users, as well as associate the users with the smart freezer, its content, and the usage behavior. Traditionally, authentication systems used to access cloud services or web servers made use of unique identifiers, that relates the users and the IoT associated devices [26]. Nevertheless, this method exposes information about the user and the devices, through the connection to the IoT service manager. More complex authentication methods, including anonymous credential systems like the Identity Mixer (Idemix) technology developed by IBM Research [27], would better protect the user privacy concerning his/her feeding habits. For example, the user and the related HighChest could for first connect to a trusted security service provider, providing anonymous credentials to connect together or with the Internet or cloud services. This would prevent the exposing of the user identity, and only the treatability of the single connections.

## 8. Conclusions

With the increased adoption of freezers into the home environment, the presence of solutions for food management is essential for meal planning and waste reduction, even more so than in refrigerators. In this paper, we have presented HighChest, an augmented household freezer designed to optimize the storage of frozen items, promote eco-efficient behavior and reduce wastage. To the best of the authors’ knowledge, no one before has dealt with the enhancement of domestic freezer capabilities. The proposed solution allows easier management of stored food, control of food conservation and feedback to remind users of expiry dates, preventing wastage. The monitoring of the chest’s status is another important aspect, allowing the prevention of abnormal functioning or a prompt intervention to ward off thawing.

We implemented functionalities so far applied only in refrigerators, overcoming limitations of the proposed methods to reach a working and reliable prototype. As our proposed system is easily scalable, it allows the addition of more devices in series controlled together in a single and integrated inventory, suitable for large contexts, especially those where it is essential to optimize resources and reduce waste, such as in distribution chains and catering services, as well as hospitals, nurseries and canteens.

The hardware architecture of our prototype is depicted in detail. We have introduced innovative services and features, such as power supply fault detection, food expiration prevention, a 3-D food localization system and an advanced interface for food waste prevention. The food management and freezer status functions are made accessible via a tablet-based user-friendly interface. We compared the most representative smart refrigerator concepts to our proposed freezer, highlighting the vast number of implemented functionalities in our system.

The storing management of our system is barcode technology-based, so the application of a code is necessary for all products that do not come from a POS network, such as leftovers and homemade products. Currently, the system is thought to provide printable barcodes applicable to its containers. In future work, the system will be equipped with a barcode printer to extend the range of traceable products to leftovers and homemade products. The idea is to provide the user with a set of barcodes that univocally identify the various food classes. For example, a barcode will be used to distinguish all products belonging to the type ‘pork’, another will correspond to ‘pasta’, and so on. The new code will consist of eight characters, with the first four corresponding to the type (e.g., Meat) and the second four identifying the subclass of food (e.g., Chicken). In order to automate the first insertion/registration, the list of these foods will be presented in the “Prefer” database at system installation.

The rigorous design methodology, described in Section 3, enabled the identification of a number of users’ requirements and relative technical specifications. Some of them were implemented and described in this work. However other recommendations are still under development. In future developments, we plan to improve the system’s energy efficiency by adapting the defrosting procedure depending on the humid air intake during the loading phase, and controlling the compressor according to internal temperature, and amount and type of products placed inside the freezer. Indeed, proper control of inner temperature using the compressor affects food quality. Perishable food (e.g., fish) requires a storage temperature that is as constant as possible. Thus, rapid and energy-expensive compressor dynamics will be enabled only in the presence of delicate products, whereas an energy-saving control strategy will be used otherwise. To improve the user experience and to comply with the IoT framework, HighChest could turn the recipe suggestion service into a personalized dietary suggestion service. This functionality should consider preferences, possible allergies, intolerances and/or diseases of the user to promote a healthy diet. To allow better and more conscious space management, a dedicated app on smartphones will be created to enable mobile access to storage information, which currently is made possible only via e-mail or SMS.

Finally, behavioral and persuasive models of interaction could be designed as a future activity, to promote eco-friendly users behaviors and a healthy dietary. The ability of technological systems to act as a persuasive agent and induce behaviors on humans has been investigated from a long time, in different domains such as marketing, military training and the health industry [28]. Sometimes, technology is used to monitor or held the people attention, to improve the teaching/learning in the educational field [29]. In particular, many commercial devices exist for virtual coaching [30] and researchers investigated a number of solutions to assess user’s stress or induce positive emotions through biofeedback [31], improve the quality of life, promote healthy life, prevent chronic disease [32] or support seniors in daily life [28]. Most of this works rely on established psychological theories [33,34] on how people may be, are being, and will be influenced through the information technology (IT) designs. HighChest could contribute in the future, as a freezer embodiment, integrated in the IoT framework, for the investigation of persuasive strategies to promote eco-friendly behaviors.

## Figures and Tables

**Table 1:** Comparison table between the reviewed smart fridge systems and the proposed work, where ‘x’ represents figure of merit.

	Content-Aware Fridge	Cloud Fridge	PerFridge	LG Smart ThinQ™ Refrigerator	HighChest
Inbound foods scanner	x	x	x		x
Content management by local and remote				x	x
Automatic retrieving of expiry date					x
Users eating habit and balanced diet assistance	x				
Smart recipe suggestion	x			x	x
Temperature control				x	x
Power saving				x	
Door status control		x	x	x	x
Compressor control				x	
Power Supply control					x
Localization system		x			x
Embedded scale					x
References	[5]	[9]	[7]	[15]	

**Table 2:** Methodology followed during the expert evaluation and user trials stages.

Operating Steps	Main Objectives	Methodology	Expected Results
1. Expert evaluation *	Assess the accessibility and dimensional requirements	Anthropometric evaluation through 3D simulation software (Makehuman™ and Rhinoceros)	Identify dimensional requirements of HighChest
	Map use phases via functional correlation analysis between use activities and the system	Task Analysis	Mapping of the system and tasks necessary for its use. This allows the highlighting of criticisms, to define what should be tested during user trials.
2. User Trials	Usability evaluation: subjective perception (satisfaction) of the system, and human–machine efficiency and effectiveness	User interviews (Contextual Inquiry [17], Observation and Thinking Aloud [18])	To collect opinions, thoughts, expectations, criticisms and intuitions useful for defining the design concept and user interface.

* The preliminary evaluation stage was meant to define the main objectives to be investigated in the user trials stage.

**Table 3:** Design phase outcomes based on assessment and user involvement stages.

	Criticisms and User Requirements	Implemented Solutions
**Food and Space Management**	- Low efficacy of containment systems (metal baskets); - Difficult to trace foods placed in the lower part of the freezer; - A system is needed for food identification and the implementation of a database to monitor expiration dates and rapidly assess the presence or absence of a specific product; - Risk of reduction of thermal performances due to prolonged opening times.	- Encourage user to organize storage by type of food, providing assistance concerning storage location during product insertion; - Develop a localization system to trace the position of stored products; - Implement a system for tracking expiration date of loaded foodstuffs; - Suggest recipes to the user based on expiring products, thus avoiding food waste; - Evaluate the effect of humid air intake at door opening on freezer performance.
**User Interface**	- Traditional appliance interfaces are confusing and some icons are barely visible; - Traditional buttons on chest freezers are somewhat sensitive to touch; - Unclear correspondence between buttons and icons; - The usability of traditional interfaces is reduced because they often use only one button for controlling several functions (one key for five functions); - It is not easy to identify the nature of alarms on traditional interfaces (e.g., power failure, temperature rise, door openings); - Appliances should provide feedback in case of faults/alarms to reduce the need for customer-service.	- Increase the size of the control panel and information icons, and consider replacing it with a touch screen monitor; - Create input commands that follow the principle of mapping, with a simple relationship between control and effect; - Avoid a single control input for multiple functions; - Implement services for operation monitoring, providing clear and dedicated alarms in the case of failures.

**Table 4:** Main characteristics of the sensors used on HighChest.

Sensor	Measure	Manufacturer	Measure range	Accuracy
CZL204E	Weight	Phidgets (Calgary, AB, Canada)	0 kg to 50 kg	±100 g
STCN75DS2F	Temperature	STMicroelectronics (Geneva, Switzerland)	−55 °C to 125 °C	±0.5 °C
HIH-5030	Humidity	Honeywell ( Morris Plains, NJ, USA)	0% RH to 100% RH	±3%
KSK-1A46-1020	Contact	Standex-Meder (Cincinnati, OH, USA)	Open/Close	-

**Table 5:** Localization errors accounted for six samples over the six compartments.

Compartments	1	2	3	4	5	6
Mean error (std) [cm] for 0.5 kg	4.69 (0.33)	5.00 (0.20)	5.81 (0.60)	7.32 (0.78)	5.42 (0.86)	5.37 (0.48)
Mean error (std) [cm] for 1 kg	4.35 (1.16)	3.45 (1.64)	3.79 (1.19)	3.39 (0.93)	3.09 (0.60)	3.52 (1.26)
Mean error (std) [cm] for 1.5 kg	4.34 (1.23)	3.21 (1.20)	2.26 (1.74)	0.95 (0.46)	1.20 (0.50)	2.59 (1.00)
Mean error (std) [cm] for 4 kg	2.62 (1.03)	2.12 (0.65)	1.64 (0.84)	5.26 (0.60)	2.67 (0.89)	2.91 (0.29)

## References

[1] Caronna S. (2011). Report on How to Avoid Food Wastage: Strategies for Improving the Efficiency of the Food Chain in the EU.

[2] FAO (2011). Global Food Losses and Food Waste—Extent, Causes and Prevention.

[3] Gaiani S. (2013). Lo Spreco Alimentare Domestico in Italia: Stime, Cause ed Impatti. Ph.D. Thesis.

[4] VISTA Brand of Commercial & Household Appliance “Technical Information”. http://blinternationaltrading.webs.com/customerinformation.htm.

[5] Gu H., Wang D. A content-aware fridge based on RFID in smart home for home-healthcare. Proceedings of the 11th International Conference on Advanced Communication Technology (ICACT 2009).

[6] Luo S., Jin J., Li J. (2009). A smart fridge with an ability to enhance health and enable better nutrition. Int. J. Multimed. Ubiquitous Eng..

[7] Rouillard J. The Pervasive Fridge. A smart computer system against uneaten food loss. Proceedings of the Seventh International Conference on Systems (ICONS2012).

[8] Noutchet A.D. (2013). Novel User Centric RFID Fridge Design. Comput. Inf. Sci..

[9] Sandholm T., Lee D., Tegelund B., Han S., Shin B., Kim B. (2014). CloudFridge: A Testbed for Smart Fridge Interactions. arXiv.

[10] Murata S., Kagatsume S., Taguchi H., Fujinami K. Perfridge: An augmented refrigerator that detects and presents wasteful usage for eco-persuasion. Proceedings of the 2012 IEEE 15th International Conference on Computational Science and Engineering (CSE).

[11] Brown P. (2012). Shelf Life Expiration Date Management. U.S. Patent.

[12] Chandran S. (2016). Intelligent (smart) cabinets, drawers and refrigerator arrays allow remote monitoring and reporting of commodities inside from the internet (mobile and web applications).

[13] Family Hub^TM^ Multi-door Fridge Freezer, 550L. http://www.samsung.com/uk/refrigerators/multi-door-rf56k9540sr/.

[14] A Close Look at LG’s Smart ThinQ LFX31995ST Refrigerator (Hands-On). https://www.cnet.com/products/lg-smart-thinq-lfx31995st-refrigerator/preview/.

[15] LG Smart ThinQ™ Refrigerator. http://www.lg.com/us/refrigerators/lg-LFX31995ST-french-3-door-refrigerator.

[16] Hollnagel E. (2012). Task analysis, why, what and how. Handb. Hum. Factors Ergonomics.

[17] Beyer H., Holtzblatt K. (1999). Contextual design. Interactions.

[18] Ericsson K.A., Simon H.A. (1985). Protocol Analysis: Verbal Reports as Data.

[19] Vezzoli C., Manzini E. (2007). Design per la Sostenibilità Ambientale.

[20] ISO 9241 (2010). ISO 9241 Part 210: Human-centred design for interactive systems. ISO 9241 Ergonomics of Humansystem Interaction.

[21] McCathie L. (2004). The Advantages and Disadvantages of Barcodes and Radio Frequency Identification in Supply Chain Management. Bachelor’s Thesis.

[22] Bøgh-Sørensen L. (2006). Recommendations for the Processing and Handling of Frozen Foods.

[23] Waide P., Lebot B., van der Sluiss S Analysis of the Efficiency of European Domestic Refrigerators 1 Year After the Energy Label. http://aceee.org/files/proceedings/1996/data/papers/SS96_Panel3_Paper21.pdf.

[24] Blumenthal J., Grossmann R., Golatowski F., Timmermann D. Weighted centroid localization in zigbee-based sensor networks. Proceedings of the IEEE International Symposium on Intelligent Signal Processing (WISP 2007).

[25] Pirbhulal S., Zhang H., Mukhopadhyay S.C., Li C., Wang Y., Li G., Zhang Y.T. (2015). An efficient biometric-based algorithm using heart rate variability for securing body sensor networks. Sensors.

[26] Camenisch J., Lysyanskaya A. (2001). An efficient system for non-transferable anonymous credentials with optional anonymity revocation. International Conference on the Theory and Applications of Cryptographic Techniques.

[27] Hu C., Li H., Huo Y., Xiang T., Liao X. (2016). Secure and Efficient Data Communication Protocol for Wireless Body Area Networks. IEEE Trans. Multi-Scale Comput. Syst..

[28] Kang Y., Tan A.H., Miao C. An adaptive computational model for personalized persuasion. Proceedings of the 24th International Joint Conference on Artificial Intelligence 2015.

[29] Santos O.C., Uria-Rivas R., Rodriguez-Sanchez M.C., Boticario J.G. (2016). An open sensing and acting platform for context-aware affective support in ambient intelligent educational settings. IEEE Sens. J..

[30] Akker H., Jones V.M., Hermens H.J. (2014). Tailoring real-time physical activity coaching systems: A literature survey and model. User Model. User Adapt. Interact..

[31] Wu W., Zhang H., Pirbhulal S., Mukhopadhyay S.C., Zhang Y.T. (2015). Assessment of biofeedback training for emotion management through wearable textile physiological monitoring system. IEEE Sens. J..

[32] Qian H., Kuber R., Sears A., Murphy E. (2011). Maintaining and modifying pace through tactile and multimodal feedback. Interact. Comput..

[33] Oinas-Kukkonen H., Harjumaa M. (2009). Persuasive systems design: Key issues, process model, and system features. Commun. Assoc. Inf. Syst..

[34] Fogg B.J. (2002). Persuasive technology: Using computers to change what we think and do. Ubiquity.

